# Exposure to Electromagnetic Fields From Smart Utility Meters in GB; Part III) On‐Site Measurements in Homes

**DOI:** 10.1002/bem.22202

**Published:** 2019-06-24

**Authors:** Carolina Calderon, Darren Addison, Nishtha Chopra, Simon Mann, Myron Maslanyj, Azadeh Peyman

**Affiliations:** ^1^ Radiation Dosimetry Department, Centre for Radiation, Chemical and Environmental Hazards Public Health England Didcot UK

**Keywords:** EMF, RF, Smart meter, ZigBee, Public exposure

Smart meters are regarded as an important step toward the development of a smart grid, with the prospect of delivering improved network efficiency and responsiveness, and playing an important role in the transition to a low‐carbon economy.

Smart meters send utility companies energy consumption data at regular intervals, eliminating the need for estimated or manual meter readings. This is done via a wide area network (WAN). The intention is also to provide consumers with real‐time information to help them understand and optimize their energy use. This is done using a home area network (HAN), consisting of an in‐home display (IHD) connected to a communication hub (CH), which coordinates the HAN communications, an electricity meter (EM), and sometimes a gas meter (GM). Smart meter devices use radiofrequency (RF) electromagnetic fields to communicate with systems both inside and outside the home and people near the equipment are exposed to these fields.

In 2012, in response to possible public concern, Public Health England (PHE) started a systematic assessment of the exposure of people to RF radiation from smart meter devices within the HAN. Part I of the project, comprised of power density measurements around smart meter devices under controlled laboratory conditions [Peyman et al., 2017], and part II, involving calculation of the specific absorption rate from exposure to smart meters [Qureshi et al., 2018], have been completed.

This letter presents the final part of this project, which aimed to provide quantitative information on exposure levels in real scenarios within homes. To quantify exposure in terms of exposure guidelines, two metrics were considered: the power per unit area of the transmitted EMF, or “power density” and the proportion of time that devices were transmitting, in terms of “duty factor.” Three source‐exposure scenarios were identified for investigation purposes: (i) all operational HAN smart meter devices located in the homes; (ii) other RF sources identified in the homes (environmental measurements); (iii) the “bank of meters” scenario, where several meters are installed in groups, designed for serving multiple‐occupancy buildings, such as blocks of flats.

With the assistance of Smart Energy GB and the UK's Department for Business, Energy & Industrial Strategy (BEIS), a convenience sample of 20 homes was accrued, across different regions in England, with customers from nine different utility companies. The sample included, where possible, different types of properties (detached, semi‐detached, terrace, flat, and bungalow) and included smart meters made by eight different manufacturers. In reporting the results of field measurements, the manufacturer's name and model of smart meter devices are not identified. The models tested are regarded as examples of typical equipment available in Great Britain. All the smart meters in this project operate the HAN using ZigBee technology at 2.4 GHz. ZigBee is a communication protocol that uses low‐power RF signals based on the Institute of Electrical and Electronics Engineers (IEEE) 802.15.4 standard for low‐rate (in data terms) wireless personal area networks [IEEE, 2011]. Only first‐generation smart meters were available at the time of the project, meaning they all followed the protocol described in the Smart Metering Equipment Technical Specifications 1 [DECC, 2014].

All properties surveyed had a CH, IHD, and smart EM. In some homes the GM was either not present or not part of the smart meter network (*n* = 4). In most properties surveyed (*n* = 14), the EM was wired directly to the CH and thus would not emit (as communications were only with the CH). In these cases, isolated power density measurements of the CH transmissions were possible. However, for the remainder of the properties (*n* = 6), both the EM and CH were adjacent to each other and transmitted at a similar rate, thus power density measurements captured the signal from both devices. For this reason, isolated power density measurements of EMs were not possible due to the proximity to the CHs. This did not affect the duty factor measurements as each device had its own ID.

Power density measurements were performed with a Narda Selective Radiation Meter 3006 (Narda STS, Pfullingen, Germany), hereafter referred as SRM, connected to a three‐axis isotropic electric field sensor, either model 3501/3 (Narda STS) with a frequency range of 75 MHz–3 GHz or model 3502/1 (Narda STS) with a frequency range of 420 MHz–6 GHz [Narda Safety Test Solutions, 2010]. The display range was 8 nW/m^2^ to 106 W/m^2^ for the 3501/3 model and 3 nW/m^2^ to 68 W/m^2^ for the 3502/1 model; the lower range corresponds to the Displayed Average Noise Level at 2.1 GHz, with a 2 MHz resolution bandwidth (RBW), lowest measurement range, and root mean square (RMS) detection. The SRM has a calibration traceable to the manufacturer's standards. The manufacturers’ quoted uncertainty for a single axis measurement was −2.4/1.9 dB for the 3501/3 model and −2.2/1.7 dB for the 3502/1 model.

The SRM has multiple recording mode options; two were used during these surveys: “Level Recorder” (LR) and “Safety Evaluation” (SE) mode. The LR mode is recommended for pulsed infrequent single‐frequency signals such as smart meter HAN transmissions, while the SE mode is recommended for quick measurements over multiple frequency channels.

LR power density measurements were made in the active Zigbee channel (identified as described below) at distances of 0.5 and 1 m from each of the smart meter devices. The chosen distances ensured measurements were made in the far field region, where measurements are more reliable, and at the same time close enough to the source for the signal to be above the noise floor of the probe. The LR mode displays peak and RMS measurements over a period of time, in this case set to 6 min, in accordance with the International Commission on Non‐Ionizing Radiation Protection (ICNIRP) guidelines [ICNIRP, 1998]. The RBW was set to 2 MHz, where the Zigbee signal is contained [Farahani, 2008; Peyman et al., 2017].

Sequential single‐axis measurements were used for the LR mode measurements (as opposed to isotropic) to ensure each electric field component of the burst was captured. This was particularly relevant for GM measurements owing to the sporadic transmissions of these particular devices (which can be as infrequent as every 30 min). The total power density was given by the sum of the orthogonal single‐axis measurements.

On arrival at each property, the active Zigbee channel was identified, by examining the field levels displayed across all 16 Zigbee channels on the SRM (on SE mode) at touching distance from one of the smart meter devices. Sniffing software and hardware were then used to capture traffic in the channel with the strongest field strength. The Wireless Local Area Network (WLAN) at 2.4 GHz occupies the same frequency band as Zigbee devices, thus any WLAN devices present in the same room as the smart meter devices were either temporarily turned off, or shielded with a Faraday bag to avoid possible swamping of the Zigbee signal.

A Zigbee network has a unique Personal Area Network Identifier (PAN ID) and each device within it will have a unique short ID. The duty factor of each smart meter device is obtained by identifying all relevant IDs and capturing all associated traffic. This was achieved using a packet sniffing system with a Zigbee network packet analyzer. The setup consisted of a Telegisis ETRX357 sensor module (Silicon Labs, Austin, TX) connected to an ISA3 adapter (Silicon Labs), whose output was analyzed by a laptop running Ember Insight Desktop network packet analyser software (Silicon Labs). The sniffer system was set to capture packets in the active Zigbee channel. It was necessary to gather traffic for a minimum of 2 h to capture at least four sets of GM transmissions. If a property did not have a smart GM, the recording time would be reduced to a minimum of 1 h 30 min.

The captured data provided detailed characteristics of the transmitted packets, including information such as time stamp, packet type, source, and destination PAN ID and short ID, and raw hexadecimal data (containing the length of the physical layer payload), all of which allowed for the calculation of the duty factor for each device. The sniffer system was also used to determine when the GM transmissions were likely to occur, and to check that a GM transmission had indeed occurred during the SRM measurements.

To identify signals from any other RF sources present in the immediate environment in each property, max‐hold RMS and average RMS power density measurements over 6 min were recorded across 33 defined frequency bands (75 MHz to 6 GHz) using the (isotropic) SE mode of the SRM (Table 1).

**Table 1:** Frequency Bands Covered in the Environmental RF Measurements

Band name	Frequency range (MHz)	Band name	Frequency range (MHz)
SAR	80–87.45	GSM 900 DL	925–959.9
FM radio broadcast	87.5–108	Space Research	960.5–1,350
Aero radio navigation	108.5–136.75	LTE SDL	1,452–1,492
Space/Maritime	137–174	GSM/LTE 1800 UL	1,710–1,785
Outside Broadcast	174–217.45	GSM/LTE 1800 DL	1,805–1,879
DAB Radio	217.5–230	DECT	1,880–1,900
TETRA UL	380–389.5	UMTS UL	1,920–1,980
TETRA DL	390–400	UMTS DL	2,110–2,170
TETRA UL	450–460	WLAN 2 GHz	2,400–2,483.5
TETRA DL	460–470	ISM	2,483.51–2,499
Broadcast TV	470–790	LTE 2600 UL	2,500–2,595
LTE 800 DL	791–821	LTE 2600 DL	2,620–2,690
LTE 800 UL	832–862	WiMAX	3,400–3,800
M‐Bus	868–870	WLAN 5 GHz A	5,150–5,350
GSM‐R UL	876–879.9	WLAN 5 GHz B	5,470–5,725
GSM 900 UL	880–915	WLAN 5 GHz C	5,795–5,871.5
GSM‐R DL	921–924.9		

DAB = digital audio broadcasting; DECT = digital enhanced cordless telecommunications; DL = down link (transmission from cell site to mobile phone), FM = frequency modulation, GSM = global system for mobile communications; GSM‐R = global system for mobile communications—railway, ISM = industrial, scientific and medical, LTE = long‐term evolution, M‐Bus = meter‐bus, SAR = search and rescue, SDL = supplemental downlink, TETRA = terrestrial trunked radio, UL = up link (transmission from mobile phone to cell site), UMTS = universal mobile telecommunications system, WiMax = Worldwide interoperability for microwave access, WLAN = wireless local area network.

Spot measurements were performed in the center of three of the most occupied rooms reported by home occupiers. The center of the probe was positioned at 1 m from the floor and at least 1 m from any other RF source. Any visible RF sources in the rooms were noted, typically WLAN access points (AP) and mobile phones/laptops, as were any nearby phone masts.

The SRM's 2 GHz WLAN band in SE mode was too broad to differentiate WLAN from smart meter signals. Thus, in two properties where both types of devices were present in the same room, an additional Zigbee LR measurement was performed in the center of the room, as well as a more detailed SE measurement focusing on the WLAN channels. These measurements allowed a rough estimate to be made of the relative proportion of WLAN and Zigbee signals in the overall RF environment.

For the bank of meters, measurements were made in a utility test facility. The facility consisted of 16 combined EM/CH devices (with CH being the sole transmitter), operating across 11 Zigbee channels (with some channel reuse with different PAN IDs), spread across three rows and over an area of around 1 m^2^. The bank of meters was mounted on a 7.5 cm‐thick wall made of wood and medium‐density fiberboard (MDF) material. Measurements were made at distances of 0.5 and 1 m from the front and from the back of the wall, with the probe positioned in the center of the bank of meters.

Each meter pair (EM/CH) was connected to a GM and IHD to simulate normal traffic conditions. The GMs in the network were located on an adjacent wall in front of the EMs, while the IHDs were located much further away and therefore unlikely to affect the measurements. Six‐minute max‐hold RMS power density measurements were carried out across all 16 Zigbee channels, using the SRM's SE isotropic mode. This mode was used because the large range of channels meant it was impractical within the time constraints of the survey to make single axis LR mode measurements. For this reason, and because the SE mode uses RMS detection, average RMS power density measurements for the bank of meters are not reported here as they lead to underestimation of the true exposure. The max‐hold RMS, on the other hand, provides a conservative estimate of power density for health risk assessment purposes. With this in mind, some caution is warranted in drawing direct comparisons between measurements made using different capturing modes.

For each channel, traffic was captured and recorded for approximately 47 min, thus ensuring that at least one GM transmission event was also captured in the data. Channel reuse meant that some of the 16 HANs shared the same channel. This was evident from the different PAN IDs reported for a given channel, and when this happened, all duty factors for that channel were combined.

The distribution of the collated LR peak power density measurements at 0.5 m from all home smart meter devices was found to be skewed close to log‐normal, with a geometric mean of 2.02 mW/m^2^ and a 95% confidence interval of (0.21–19.83) mW/m^2^. The distribution for the LR 6‐min RMS power density was more irregular due to differences in duty factor across types of smart meter devices.

**Figure 1:**
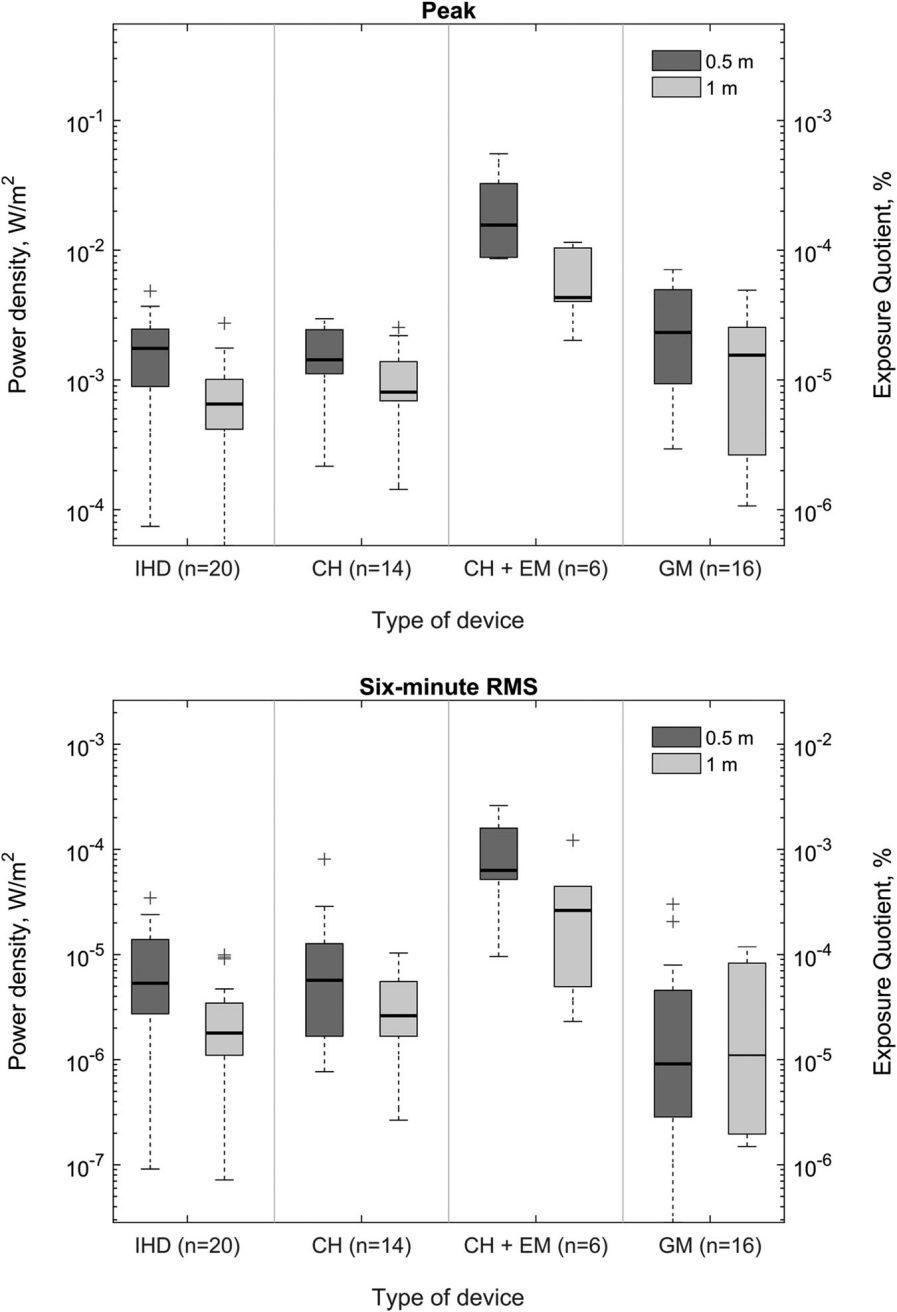
Root mean square (RMS) (bottom) and peak (top) power density over 6 min for the various smart meter devices at two distances. The secondary axis shows values in terms of percentage of the ICNIRP public reference level (10 W/m^2^ for 6 min RMS average, 10,000 W/m^2^ for maximum). The bottom and top boundaries of the shaded boxes correspond to the 25th and 75th percentile, while the line within the box shows the median. The whiskers extend to the most extreme data points not considered outliers (an outlier is a value that is more than 1.5 times the interquartile range), and the outliers are plotted individually using the “+” symbol. The values in brackets show the sample size for each group at 0.5 m.

**Figure 2:**
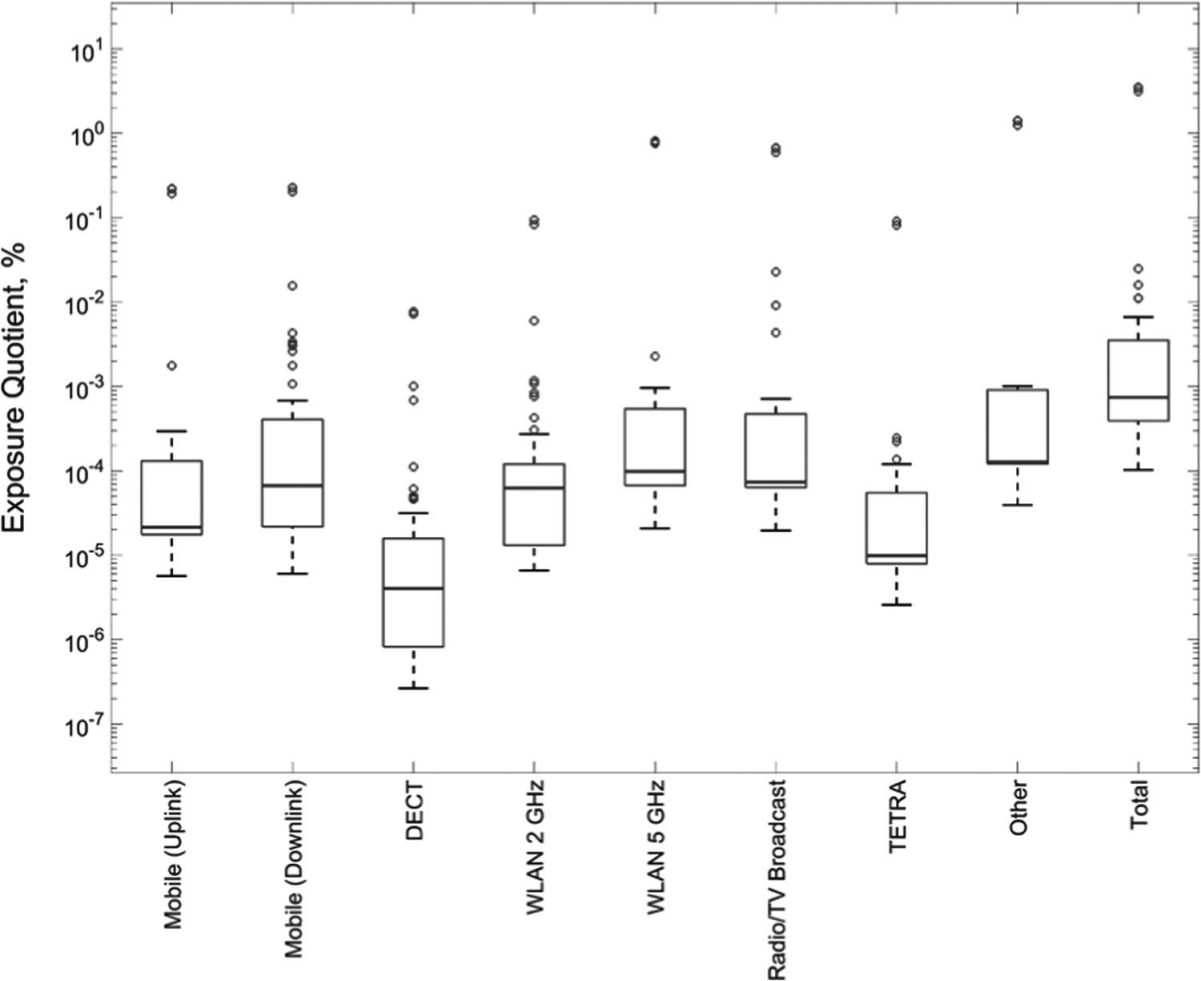
Exposure (6‐min average) as a percentage of International Commission on Non‐Ionizing Radiation Protection (ICNIRP) public reference levels for various environmental radiofrequency (RF) sources (*N* = 61). “Mobile uplink” and “Mobile downlink” groups all bands related to uplink and downlink mobile phone communications respectively: global system for mobile communications (GSM) (900, 1,800 MHz), GSM–railway (GSM‐R), universal mobile telecommunications system (UMTS), Long Term Evolution (LTE) (800, 1,800, and 2,600 MHz). “Radio/TV broadcast” groups frequency modulation (FM), digital audio broadcasting (DAB), and TV broadcasts. Terrestrial trunked radio (TETRA) groups uplink and downlink TETRA (band A and B). “Other” groups: search and rescue (SAR), Aero Radio Navigation, Space Maritime, Outside Broadcast, Space Research, ISM, WiMAX, and M‐Bus. “Total” stands for total exposure quotient and includes all sources picked up during the environmental measurements. The bottom and top boundaries of the boxes correspond to the 25th and 75th percentile, while the line within the box shows the median. The whiskers extend to the most extreme data points not considered outliers (an outlier is a value that is more than 1.5 times the interquartile range), and the outliers are plotted individually using the “o” symbol.

For peak and RMS power density, there was no statistical difference between the IHDs, CHs, and GMs (*P* > 0.39) for both sets of distances measured (Fig. 2). In fact, the median peak power density values were very similar. Combined EM and CH power densities (peak and RMS) were significantly higher (*P* < 0.004) than other isolated devices (Fig. 1), albeit the small sample size. The maximum 6‐min averaged RMS power density across all the devices measured was found to be 0.26 mW/m^2^, which is less than 0.003% of the ICNIRP general public reference level.

The range of duty factors observed for CH, IHD, and EM were 0.01–0.92%, 0.01–1.19%, and 0.13–0.27%, respectively. Across the six properties where the EM was transmitting next to the CH, the combined duty factor was found to be less than 0.8%. As expected, the GM transmissions were much less frequent (*P* < 1e^−4^) than those for IHDs and CHs, with a median duty factor of 0.002% and a 90th percentile of 0.004%. During the surveys it was noted that some of the GM transmissions occurred more frequently than every 30 min (as frequently as every 7 min); however, the duty factors were still much lower than those from other devices (<0.01%).

Because CHs are the coordinators of the network, it might be expected that their duty factor would be related to the duty factor of other devices in the network, and may be even higher. Indeed, a linear stepwise regression showed that the duty factor from CHs was mainly dependent on the duty factor of the IHD and EM although it was not found to be significantly higher than these. For example, the highest duty factor observed across all CHs was measured in the same home where the largest IHD duty factor was found. The CH duty factor was not, however, significantly (*P* > 0.6) dependent on the GM duty factor most likely because of the relatively infrequent transmissions.

A small proportion of the packets captured by the sniffing software did not have source information and therefore could not be allocated to a particular smart meter device. This happened mostly when either the sniffing system could not lock onto the signal of the captured packet (due to a weak signal or presence of noise), or because it captured two simultaneous packets. Unidentified transmissions were on average less than 8% of the duty factor. The maximum recorded impact of unidentified transmission was 20%, and this was for a CH in one particular property.

Environmental measurements were mainly made in living rooms (31%), kitchens (31%), and main bedrooms (16%). The 6‐min average (RMS) results, shown in Figure 2 as a percentage of the ICNIRP guidelines, have been collated into the following groups to help interpretation: mobile telecommunication uplink (phone), mobile telecommunication downlink (base stations), cordless phones (Digital Enhanced Cordless Telecommunications), WLAN (2 and 5 GHz), Radio and Television (TV) broadcast, Terrestrial Trunked Radio (TETRA), and Other. The total max‐hold RMS power density across all bands in the environmental measurements had a skewed distribution with a geometric mean of 0.55 mW/m^2^ and a 95% confidence interval of (0.01–27.85) mW/m^2^.

The WLAN 2 GHz band can contain contributions from both WLAN and Zigbee devices present in the environment. However, in only two of the 61 rooms measured was there a smart meter device (an IHD) in the same room as the AP. Additional measurements in the center of these rooms showed that WLAN signal was at least four times higher than the Zigbee signal.

**Figure 3:**
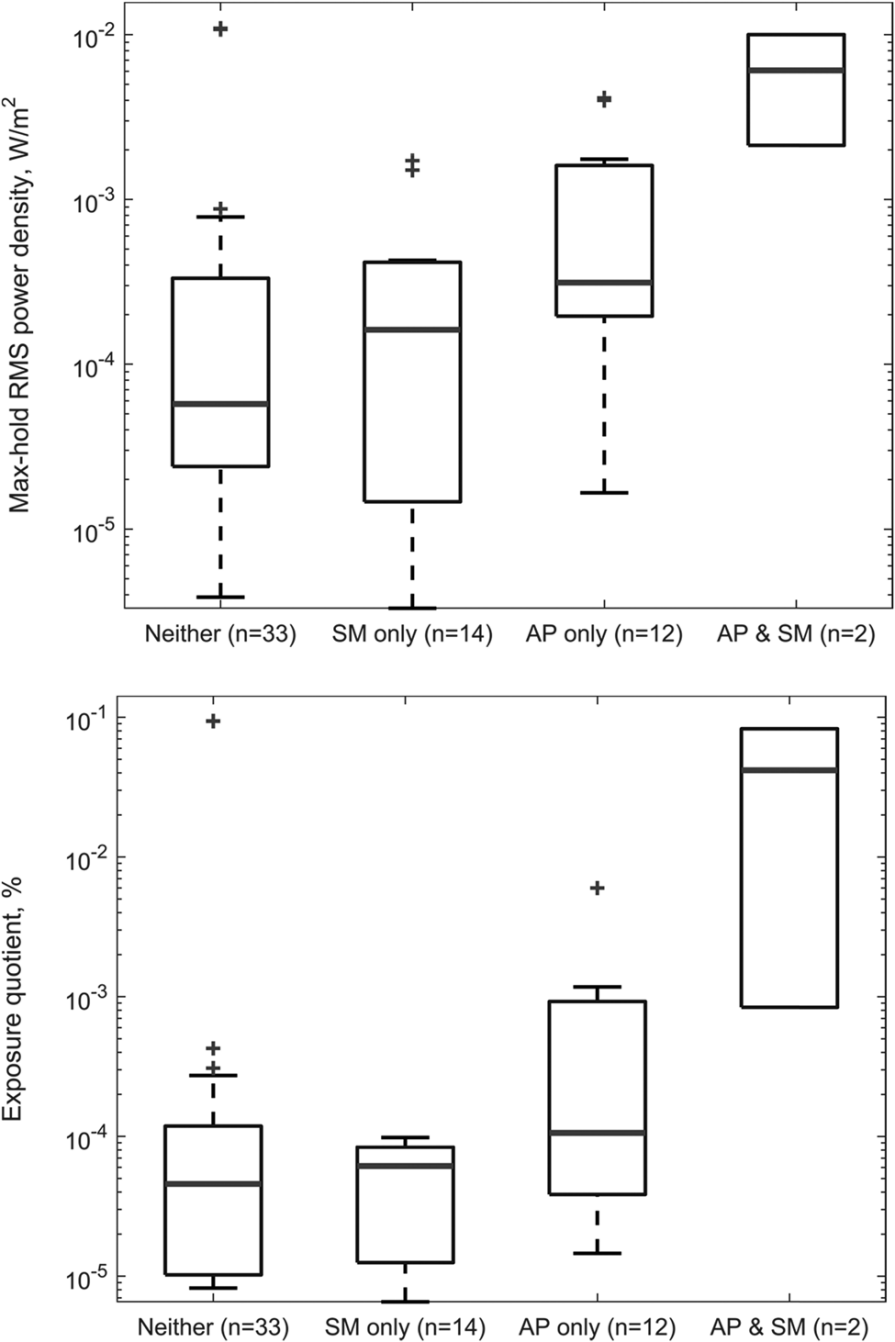
Environmental measurements in the center of rooms with and without a smart meter (SM) or Wireless Local Area Network (WLAN) access point (AP) present (*n* is the number of rooms where measurements have taken place).

Although not statistically significant (because of the low sample numbers), power density seems to be higher when both access points and smart meter devices are both present in a room followed by when only APs are present, and then when only smart meter devices are present (Fig. 3). It should be noted that the group labeled “Neither” in Figure 3 includes rooms where there were other WLAN‐enabled devices, such as mobile phones and laptops (but were not necessarily transmitting in this band).

For the bank of meters, the highest value recorded was 0.5 m away from the front of the bank. Power densities recorded behind the wall were generally lower; this is probably due to the directional nature of the transmitting antennas [Peyman et al., 2017] rather than any attenuation of the materials used in the construction of the wall. Power density measurements at 1 m behind the wall were higher than the 0.5 m values. One explanation could be contributions from reflections from another wall or from other uncontrolled meters in the test facility, though this cannot be verified.

Max‐hold RMS power densities for individual channels ranged from 0.02 to 3.59 mW/m^2^, while combined power densities ranged from 1.4 to 12.1 mW/m^2^ (across the four different measurement locations), the latter being less than 0.12% of the recommended ICNIRP public reference level.

Duty factors were found to be less than 5.2% when all active channels were combined.

Power densities and duty factors were assessed for smart meter devices installed in 20 homes across England. The maximum 6‐min averaged RMS power density for all the devices measured in the homes was found to be 0.26 mW/m^2^ at 0.5 m, which is less than 0.003% of the ICNIRP general public reference level. All the duty factors measured in the homes were less than 1.2%. In addition, the geometric mean of the peak power density from smart meters at 0.5 m in the homes was found to be very similar to that measured under a controlled laboratory condition [Peyman et al., 2017]. Maximum 6‐min RMS power densities, which are affected by duty factor, were 60 times lower than the maximum peak power density observed in the laboratory.

In the case of the bank of meters, combined max‐hold (over 6 min) RMS power density from the 16 meters yielded a value of 12.1 mW/m^2^ at 0.5 m, which is less than 0.12% of the recommended ICNIRP reference level, and the combined duty factor was less than 5.2%.

The results of environmental measurements of RF sources in homes suggest that background exposure from the 2 GHz band (which includes WLAN and Zigbee) is similar or lower to common sources (e.g., mobile phone communications). It is not straightforward to isolate the contribution of smart meter devices to the overall exposure in a home because Zigbee signals are embedded in the same frequency band as WLAN devices present in the environment. Power densities seem to be higher when both WLAN and smart meter devices are present in a room, followed by when only WLAN devices are present, and then when only smart meter devices are present. On the two occasions when a smart meter and WLAN device were in the same room, additional measurements in the center of rooms showed that WLAN signal was at least four times higher than that from the smart meter device. It is also notable that smart meter devices generally have smaller duty factors (1.2% being the highest measured) compared with those from WLAN devices, reported to be up to 12% for access points [Khalid et al., 2011].
